# Bifunctional Role of CrkL during Bone Remodeling

**DOI:** 10.3390/ijms22137007

**Published:** 2021-06-29

**Authors:** Jung Ha Kim, Kabsun Kim, Inyoung Kim, Semun Seong, Hyun Kook, Kyung Keun Kim, Jeong-Tae Koh, Nacksung Kim

**Affiliations:** 1 Department of Pharmacology, Chonnam National University Medical School, Gwangju 61469, Korea; kjhpw@hanmail.net (J.H.K.); kabsun@hanmail.net (K.K.); doll517@naver.com (I.K.); iamsemun@chonnam.edu (S.S.); kookhyun@chonnam.ac.kr (H.K.); kimkk@chonnam.ac.kr (K.K.K.); 2 Hard-Tissue Biointerface Research Center, School of Dentistry, Chonnam National University, Gwangju 61186, Korea; jtkoh@chonnam.ac.kr; 3 Department of Pharmacology and Dental Therapeutics, School of Dentistry, Chonnam National University, Gwangju 61186, Korea

**Keywords:** osteoclast, osteoblast, RANKL, coupling signal, CrkL, bone homeostasis

## Abstract

Coupled signaling between bone-forming osteoblasts and bone-resorbing osteoclasts is crucial to the maintenance of bone homeostasis. We previously reported that v-crk avian sarcoma virus CT10 oncogene homolog-like (CrkL), which belongs to the Crk family of adaptors, inhibits bone morphogenetic protein 2 (BMP2)-mediated osteoblast differentiation, while enhancing receptor activator of nuclear factor kappa-B ligand (RANKL)-induced osteoclast differentiation. In this study, we investigated whether CrkL can also regulate the coupling signals between osteoblasts and osteoclasts, facilitating bone homeostasis. Osteoblastic CrkL strongly decreased RANKL expression through its inhibition of runt-related transcription factor 2 (Runx2) transcription. Reduction in RANKL expression by CrkL in osteoblasts resulted in the inhibition of not only osteoblast-dependent osteoclast differentiation but also osteoclast-dependent osteoblast differentiation, suggesting that CrkL participates in the coupling signals between osteoblasts and osteoclasts via its regulation of RANKL expression. Therefore, CrkL bifunctionally regulates osteoclast differentiation through both a direct and indirect mechanism while it inhibits osteoblast differentiation through its blockade of both BMP2 and RANKL reverse signaling pathways. Collectively, these data suggest that CrkL is involved in bone homeostasis, where it helps to regulate the complex interactions of the osteoblasts, osteoclasts, and their coupling signals.

## 1. Introduction

Bone is a complex and dynamic tissue, and it undergoes continuous renewal via bone remodeling processes to maintain appropriate bone mass and quality [1]. These continuous processes of synthesis and destruction are fine-tuned by an equilibrium between bone-forming osteoblasts and bone-resorbing osteoclasts [2,3]. In addition to the inherent functions of osteoblasts and osteoclasts, they contribute to each other’s functions via direct and indirect communication to maintain bone homeostasis [4,5,6,7]. Moreover, osteoblasts can influence osteoclastic bone resorption by producing osteoclast regulatory factors, such as macrophage colony-stimulating factor (M-CSF), receptor activator of nuclear factor kappa-B ligand (RANKL), Fas ligand, complement component 3a, and semaphorins [5,8,9,10,11,12,13,14,15,16]. Additionally, various osteoclast-derived factors, such as those released from the matrix, secreted from the osteoclast, and expressed on the cell membrane, can also influence the osteoblast differentiation and function [16,17]. For example, matrix-derived factors, such as transforming growth factor β, BMP2, and insulin-like growth factors, which are released from osteoclastic bone resorption sites on the bone surface, stimulate the differentiation of the osteoblast progenitors. Osteoclasts also secrete products, such as BMP6, sphingosine-1-phosphate, and Wnt-10b to promote osteoblast precursor recruitment and differentiation. In addition, osteoclast membrane-bound factors, such as ephrin B2 and semaphorin D, are expected to support various interactions between the osteoclasts and mature osteoblasts promoting their osteoblastic activity [17]. The communication between osteoclasts and osteoblasts is often the cause of the side effects of the presently available antiresorptive agents and anabolic agents for the treatment of bone disease [18,19,20,21,22]. Therefore, a more detailed understanding of the cellular and molecular mechanisms of bone remodeling is necessary to identify and explore new therapeutic targets for bone disorders.

The cytoplasmic adaptor CrkL (v-crk avian sarcoma virus CT10 oncogene homolog-like) belongs to the Crk family of adaptors and is comprised of a single N-terminal Src homology 2 (SH2) domain and two consecutive SH3 domains (nSH3 and cSH3). CrkL is ubiquitously expressed in most tissues and exhibits several biological functions, such as adhesion, proliferation, migration, and survival [23,24,25,26]. This adaptor protein can function via an interaction between its own SH2 or SH3 domain and numerous adaptor proteins, including paxillin, p130Cas, p120 c-cbl, insulin receptor substrate proteins, STAT5, and PI3K [25,26,27,28,29,30,31,32,33,34,35,36,37,38,39,40]. We previously found that CrkII, another adaptor in the Crk family, plays a pivotal role in osteoclast and osteoblast differentiation [41,42]. CrkII enhances osteoclast differentiation by activating Rac1, whereas it inhibits osteoblast differentiation via JNK activation [41,42]. Furthermore, CrkII and CrkL exhibit overlapping functions in certain processes, including osteoclast and osteoblast differentiation, as they share several binding partners, due to remarkable homology between them. In contrast, several studies have reported that Crk proteins exhibit separate functions, notably during development [43,44]. We previously reported that CrkL and CrkII show redundant function during osteoclast and osteoblast differentiation, as CrkL is a distinct gene transcribed from the CrkL locus but not Crk locus. The role of CrkL in communication between osteoclasts and osteoblasts during bone remodeling processes remains unknown; therefore, in the present study, we thoroughly investigated the role of CrkL during bone remodeling by considering various aspects.

## 2. Results

### 2.1. CrkL Has a Positive and Negative Effect on Osteoclast and Osteoblast Differentiation, Respectively

**Figure 1:**
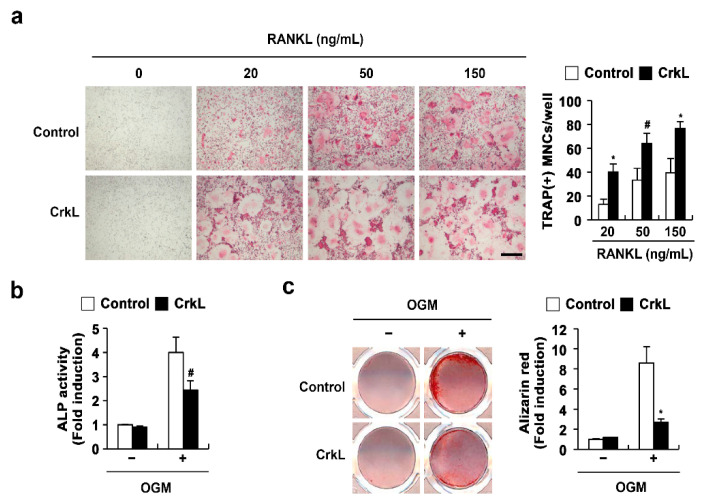
CrkL enhances osteoclast differentiation, while it inhibits osteoblast differentiation. (**a**) TRAP staining images. Osteoclast differentiation of BMMs overexpressing the control or CrkL retrovirus following treatment with M-CSF, or M-CSF and RANKL (left panel). Number of TRAP-positive multinuclear cells (right panel). Bar: 200 µm. (**b**,**c**) Primary osteoblast precursors overexpressing the control or CrkL retrovirus were cultured in OGM. (**b**) ALP activity assay. (**c**) Alizarin red staining images (left panel). Quantification of alizarin red staining intensities (right panel). # *p* < 0.05; * *p* < 0.01 as compared with the controls.

We previously reported that CrkL and CrkII exhibit overlapping functions in osteoclasts and osteoblasts. In the present study, to confirm the roles of CrkL in osteoclasts and osteoblasts, we determined the effects of retrovirus-mediated overexpression of CrkL in bone marrow-derived monocyte/macrophage lineage cells (BMMs) and primary osteoblasts. The formation of large multinucleated osteoclasts, induced by RANKL, was significantly enhanced in BMMs overexpressing CrkL compared to that in control (Figure 1a); however, alkaline phosphate (ALP) activity and bone mineralization induced by osteogenic media (OGM) was significantly inhibited in osteoblasts overexpressing CrkL (Figure 1b). These results confirmed that CrkL upregulates RANKL-mediated osteoclast differentiation, while it downregulates osteoblast differentiation and function.

### 2.2. CrkL Indirectly Inhibits Osteoclast Differentiation by Regulating RANKL (Tnfsf11) Expression

**Figure 2:**
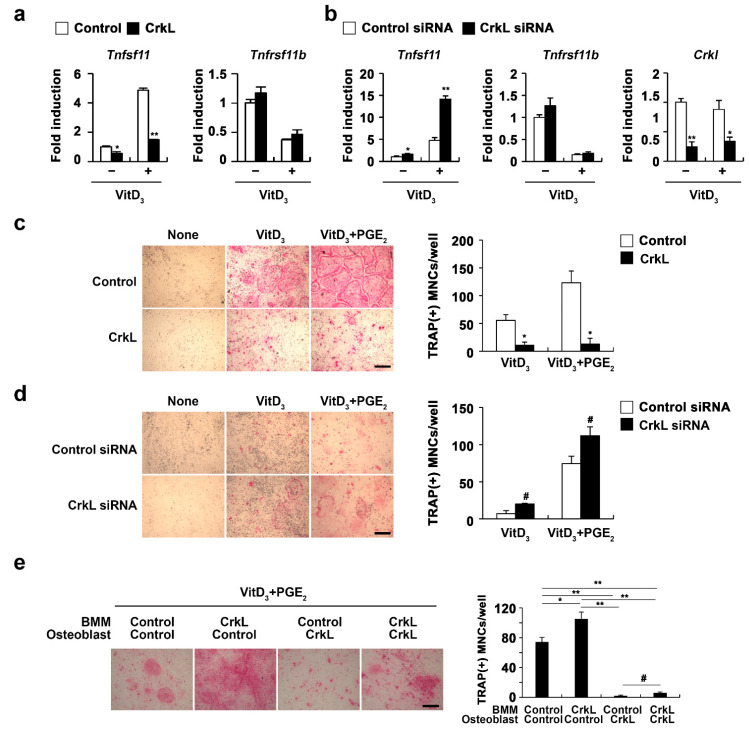
CrkL attenuates RANKL expression. (**a**) Primary osteoblast precursors overexpressing the control or CrkL retrovirus in the presence or absence of Vit D_3_. mRNA expression of the indicated genes. (**b**) Primary osteoblast precursors were transfected with control or CrkL siRNAs in the presence or absence of Vit D_3_. mRNA expression of the indicated genes was assessed by real-time PCR. (**c**) Primary osteoblast precursors overexpressing the control or CrkL retrovirus were cocultured with BMMs and Vit D_3_ in the presence or absence of PGE_2_. TRAP staining images (left panel) where used to enumerate the number of TRAP-positive multinuclear cells (right panel). Bar: 200 µm. (**d**) Primary osteoblast precursors transfected with control or CrkL siRNAs and cocultured with BMMs and Vit D_3_ in the presence or absence of PGE_2_. TRAP staining images (left panel) where used to enumerate the number of TRAP-positive multinuclear cells (right panel). Bar: 200 µm. (**e**) Osteoclast differentiation in BMMs, cocultured with osteoblasts, treated as indicated in the presence of Vit D_3_ and PGE_2_. TRAP staining images (left panel) where used to enumerate the number of TRAP-positive multinuclear cells (right panel) in each sample. Bar: 200 µm. # *p* < 0.05; * *p* < 0.01; ** *p* < 0.001 as compared with the controls.

Osteoblasts produce RANKL and osteoprotegerin (OPG), that contribute to bone homeostasis via the regulation of osteoclastogenesis [15]. We further examined whether CrkL is associated with osteoblast-mediated osteoclast differentiation. Overexpression of CrkL in osteoblasts significantly inhibited the mRNA expression of *Tnfsf11* in both nonstimulated and 1,25 (OH)_2_ vitamin D_3_ (Vit D_3_)-stimulated osteoblasts without affecting the OPG (*Tnfrsf11b*) expression (Figure 2a). Conversely, downregulation of CrkL by siRNA led to an increase in the mRNA levels of *Tnfsf11* (Figure 2b). To functionally validate the role of CrkL in osteoblastic RANKL expression, we cocultured the osteoblasts with osteoclast precursor cells. The coculture of osteoblasts overexpressing or downregulating CrkL with osteoclast precursor cells displayed decreased or increased osteoclast formation, in contrast to each control osteoblast culture (Figure 2c,d).

To further assess the role of CrkL in osteoblast-mediated osteoclast differentiation, osteoblast and osteoclast precursor cells were transduced with control or CrkL retrovirus, as illustrated in Figure 2e, and then cocultured in the presence of vitamin D_3_ (Vit D_3_) and prostaglandin E_2_ (PGE_2_). Interestingly, a significant decrease in osteoclast formation was observed when BMMs overexpressing CrkL were cocultured with osteoblasts overexpressing CrkL, compared to the control (Figure 2e). These results indicated that CrkL inhibits osteoblast-mediated osteoclast differentiation by blocking RANKL expression, though it increases RANKL-mediated osteoclast differentiation in osteoclast precursor cells.

### 2.3. CrkL Inhibits RANKL-Mediated Osteoblast Differentiation

**Figure 3:**
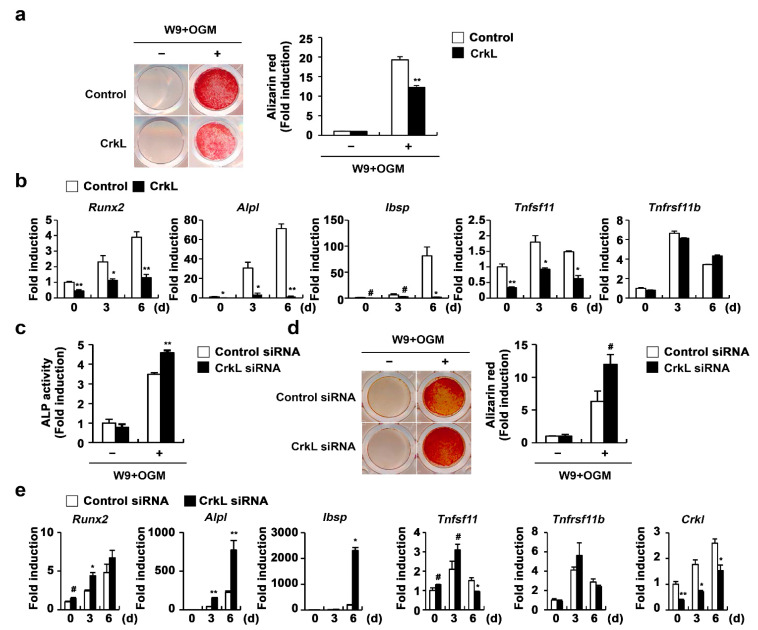
CrkL inhibits RANKL–RANK reverse signaling pathway. (**a**,**b**) Primary osteoblast precursors overexpressing the control or CrkL retrovirus were cultured in OGM supplemented with W9. (**a**) Alizarin red staining images (left panel) allowed for the quantification of the alizarin red staining intensities (right panel) under each condition. (**b**) mRNA expression of the indicated genes. (**c**–**e**) Primary osteoblast precursors transfected with control or CrkL siRNAs were cultured in OGM with W9. (**c**) ALP activity assay. (**d**) Alizarin red staining images (left panel). Quantification of alizarin red staining intensities (right panel). (**e**) mRNA expression of the indicated genes. # *p* < 0.05; * *p* < 0.01; ** *p* < 0.001 as compared with the controls.

Recently, it has been reported that vesicular receptor activator of nuclear factor kappa-B (RANK) secreted from mature osteoclasts activates RANKL reverse signaling in osteoblasts and enhances osteoblast differentiation [45]. Moreover, the W9 peptide, which is known to bind RANKL and inhibits RANKL-induced osteoclast differentiation in vitro, also binds RANKL on osteoblasts and promotes osteoblast differentiation, presumably via RANKL reverse signaling [46,47,48]. Therefore, we tested the effects of CrkL on W9-induced osteoblast differentiation, to evaluate whether the inhibition of RANKL expression in osteoblasts caused by CrkL affects osteoblast differentiation by regulating RANKL reverse signaling. As illustrated in Figure 3, overexpression of CrkL in osteoblasts significantly inhibited W9-induced bone mineralization. Consistent with bone mineralization, expression of typical osteogenic marker genes, including Runx2, alkaline phosphatase (*Alpl*), and bone sialoprotein (*Ibsp*), was significantly inhibited by CrkL overexpression (Figure 3a,b). In contrast, downregulation of endogenous CrkL expression markedly increased the ALP activity, bone mineralization, and expression of typical osteogenic marker genes (Figure 3c–e). Collectively, these results indicate that osteoblastic CrkL regulates osteoblast differentiation by inhibiting both BMP2 signaling and RANKL reverse signaling.

### 2.4. CrkL Inhibits RANKL Expression via Interaction with Runx2

**Figure 4:**
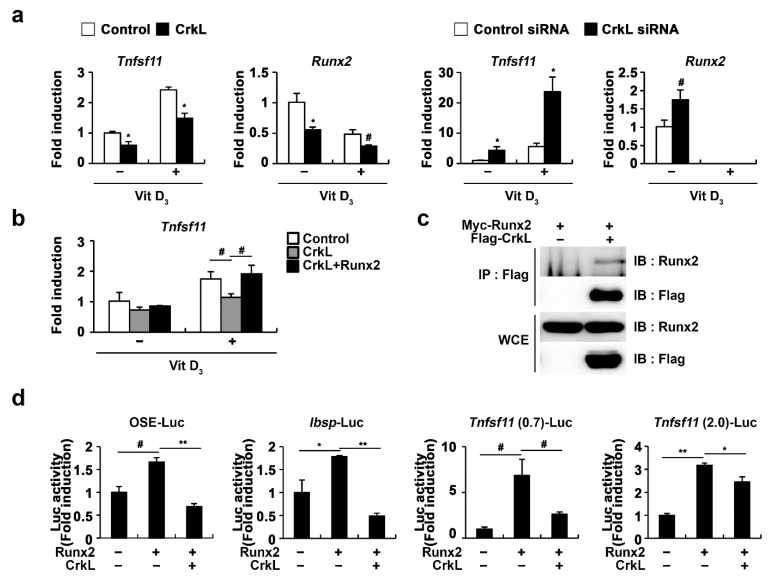
CrkL inhibits Runx2 transcriptional activity. (**a**) Primary osteoblast precursors overexpressing control or CrkL retrovirus and primary osteoblast precursors transfected with control or CrkL-siRNAs were cultured in the presence or absence of Vit D_3_. (**b**) Primary osteoblast precursors overexpressing control, CrkL or CrkL and Runx2 were cultured in the presence or absence of Vit D_3_. mRNA expression of *Tnfsf11*. (**c**) Co-immunoprecipitation assays in Runx2 or Runx2 and CrkL-transfected HEK-293T cells. (**d**) Luciferase assay of HEK-293T cells transfected with various expression plasmids. # *p* < 0.05; * *p* < 0.01; ** *p* < 0.001 as compared with the controls.

As Runx2 has been implicated in RANKL expression in various cell types, such as prostate cancer cells, vascular smooth muscle cells, and osteoblasts [49,50,51,52], we further examined whether CrkL-inhibited *Tnfsf11* expression is involved in the regulation of Runx2 expression. As illustrated in Figure 4a, Runx2 expression controlled by CrkL was similar to that of *Tnfsf11*. Moreover, the expression of *Tnfsf11* as well as that of Runx2 was suppressed when CrkL was overexpressed, whereas it was increased when CrkL was downregulated. Moreover, inhibition of *Tnfsf11* expression by CrkL was rescued by the forced expression of Runx2 (Figure 4b). In order to gain a deeper insight into the mechanisms underlying the inhibition of *Tnfsf11* expression by CrkL, we examined whether CrkL directly interacts with Runx2. Coimmunoprecipitation revealed the direct interaction between CrkL and Runx2 in HEK-293T cells (Figure 4c). Furthermore, Runx2 induced the expression of 6XOSE (Runx2 DNA-binding elements), *Ibsp*, and *Tnfsf11* promoter reporter, and its effects were significantly inhibited by CrkL (Figure 4d). Collectively, these results indicated that the CrkL-induced decreased expression of RANKL is mediated via the suppression of Runx2 transcriptional activity.

### 2.5. Downregulation of CrkL Can Protect RANKL-Induced Bone Loss In Vivo

**Figure 5:**
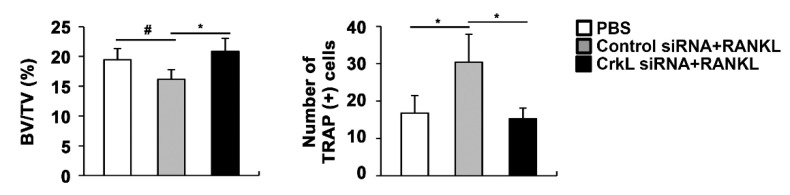
CrkL knockdown prevents RANKL-induced bone loss. Bone volume to tissue volume (BV/TV) and the number of TRAP-positive cells in the murine calvarial model treated with PBS, RANKL and control, or CrkL siRNA. # *p* < 0.05; * *p* < 0.01 as compared with the controls.

Eventually, we assessed the local administration of siRNA, targeting CrkL, in a mouse calvaria model. In microcomputed tomography (µCT) analyses, the injection of RANKL to the calvaria led to a significant decrease in bone mass. RANKL-induced bone loss was attenuated by the local administration of CrkL siRNA (Figure 5). These results indicated that CrkL may be a potential target in the development of new therapeutics for bone diseases.

## 3. Discussion

In the present study, we demonstrated that CrkL can be involved in regulating precise bone remodeling by affecting osteoclast differentiation, osteoblast differentiation, and osteoclast–osteoblast communication. The ectopic expression of CrkL in osteoclast precursor cells enhanced osteoclast differentiation and function, whereas that in osteoblasts inhibited osteoblast differentiation and function. Moreover, the inhibition of RANKL production, by the ectopic expression of CrkL in osteoblasts, hampered osteoclast-mediated osteoblast differentiation, as well as osteoblast-mediated osteoclast differentiation. Collectively, CrkL is remarkably involved in bone remodeling, and can lead to bone loss, by acting directly on osteoclasts and osteoblasts, while inhibiting both bone resorption and bone formation, by acting indirectly by regulating osteoclast–osteoblast communication.

In a previous study, we revealed that CrkII increased osteoclast differentiation via Rac1 activation and reduced osteoblast differentiation by regulating JNK activation; moreover, we demonstrated that CrkII and CrkL exhibit similar functions in osteoclast and osteoblast differentiation [41,42]. According to the results of the present study, the new function of CrkL in osteoblasts—the downregulation of *Tnfsf11* expression—is due to the inhibition of Runx2 transcriptional activities caused by the formation of a complex of Runx2 and CrkL. Runx2 acts as a master transcription factor for osteoblast differentiation, which transactivates numerous essential genes in osteogenesis, such as those encoding *Sp7*, *Alpl*, *Col1*, *Ibsp*, and *Bglap* [53,54]. Notably, Runx2 is responsible for osteoblast differentiation because bone formation is inhibited, due to the arrest of osteoblast differentiation in mice subjected to a targeted disruption of Runx2 [53]; however, the mechanisms involved in osteoclast differentiation, regulated by Runx2 in osteoblasts, remain unclear. Bone formation and osteoclasts were completely absent in *Runx2*-deficient mice, in which OPG and M-CSF were normally expressed, but RANKL was significantly less expressed [50]. Transgenic mice overexpressing Runx2 in osteoblasts revealed a dramatic increase in osteoclast differentiation and bone resorption [49]. Furthermore, the 0.7-kb 5′-flanking region of the *Tnfsf11* promoter contains two putative Runx2 binding sites [55]; however, whether Runx2 simulates *Tnfsf11* expression in osteoblasts to support osteoclast differentiation remains controversial. A previous study reported that neither Runx2 nor the dominant negative form of Runx2 expression significantly affect *Tnfsf11* expression in a stromal/osteoblastic cell line, UAMS-32 [56]. In contrast, Runx2 has been shown to directly bind to the promoter and upregulate *Tnfsf11* expression in calcifying smooth muscle cells [52]. Consistent with the latter results, we confirmed that Runx2 increased *Tnfsf11* transcription. CrkL interacts with Runx2 to inhibit Runx2 transcriptional activity, thereby suppressing the autoregulation of Runx2 and the expression of Runx2 target genes, including *Tnfsf11*. Presumably, CrkL functions primarily via the inhibition of Runx2 signaling pathway in osteoblasts. Moreover, it decreases Runx2 expression and subsequently attenuates osteoblast differentiation or osteoblast-mediated osteoclast differentiation. Furthermore, CrkL reduces the *Tnfsf11* expression, which is successfully recovered by Runx2 overexpression. As we previously reported that CrkII inhibits osteoblast differentiation by regulating JNK activation, we assumed that CrkL, similar to CrkII, also functions by regulating the JNK signaling pathway in osteoblasts. Unexpectedly, the blocking of JNK activation did not affect the *Tnfsf11* expression controlled by CrkL. Therefore, CrkII and CrkL affect the osteoblast function by mainly regulating the Runx2 signaling pathway; in particular, they inhibit osteoblast differentiation, partly by activating the JNK signaling pathway.

RANKL–RANK signaling pathway is a crucial factor in the coupling of osteoblasts and osteoclasts during bone remodeling. Osteoblasts secrete RANKL to support osteoclast differentiation. RANKL–RANK forward signaling pathway induces osteoclast differentiation and bone resorption. Meanwhile, mature osteoclasts secrete vesicular RANK, which binds osteoblastic RANKL and triggers RANKL–RANK reverse signaling pathway to stimulate osteoblast differentiation and bone formation [45]. As Runx2 activation is required for RANKL–RANK reverse signaling pathway-mediated bone formation, CrkL crucially participates in bone remodeling processes, by inhibiting the RANKL–RANK reverse signaling pathway-mediated bone formation.

The coupling of osteoblasts and osteoclasts during bone remodeling induces unexpected side effects of bone disease treatments currently in use [18,19,20,22]. Considering the bifunctional properties of CrkL, we tested whether CrkL could serve as a potential therapeutic target in the treatment of bone diseases. Our results indicated that CrkL knockdown can protect RANKL-induced bone loss in vivo; moreover, the net effect of two opposing qualities of CrkL in vivo is biased toward promoting bone resorption during bone remodeling. Although CrkL knockdown might promote osteoblast-dependent osteoclast differentiation, it contributes to bone formation by activating bone formation signaling and RANKL reverse signaling in osteoblasts and inhibiting RANKL forward signaling in osteoclast precursors. Therefore, CrkL is a potential therapeutic target for diseases related to bone loss. However, since our local and transient calvarial model is not sufficient to elucidate the precise physiological or pathological role of CrkL in vivo, further evaluations, possibly using transgenic mice with engineered CrkL expression, are required.

## 4. Materials and Methods

### 4.1. Reagents

Recombinant human BMP2 was purchased from Cowellmedi (Busan, Korea). Alizarin Red, β-glycerophosphate, PGE_2_, and Vit D_3_ were purchased from Sigma-Aldrich (St. Louis, MO, USA). Ascorbic acid was purchased from Junsei Chemical (Tokyo, Japan). Recombinant hRANKL and hM-CSF were purified from BL21.

### 4.2. Osteoclast Differentiation

Murine bone marrow cells were isolated by flushing the bone marrow of tibiae and femurs of 6–8-week-old mice with α-minimal essential medium (MEM) (HyClone Laboratories, Logan, UT, USA) and followed by red blood cell lysis. Thereafter, the isolated bone marrow cells were cultured with α-MEM containing 10% FBS and 30 ng/mL M-CSF for 3 days. Nonadherent cells were removed, and the remaining adherent cells, i.e., BMMs, were used as osteoclast precursors. BMMs were transduced with pMX-IRES-EGFP (control) or CrkL retrovirus, or transfected with control or CrkL siRNAs, depending on the experimental conditions. Transduced or transfected BMMs were cultured in M-CSF (30 ng/mL) or M-CSF and RANKL (20–150 ng/mL) for 3 days. For coculture experiments, BMMs were cultured with osteoblasts in the presence of Vit D_3_ (10^−8^ M) or Vit D_3_ (10^−8^ M) and PGE_2_ (10^−7^ M) for 6 days. Cultured cells were fixed and stained with tartrate-resistant acid phosphatase (TRAP) solution. TRAP-positive cells with more than three nuclei were counted as osteoclasts.

### 4.3. Osteoblast Differentiation

Primary osteoblast precursor cells were obtained from the enzymatic lysis of the skulls of neonatal ICR mice using 0.1% collagenase (Life Technologies, Carlsbad, CA, USA) and 0.2% dispase II (Roche Diagnostics GmbH, Mannheim, Germany). Primary osteoblasts were transduced with pMX-IRES-EGFP (control) or CrkL retrovirus, or transfected with control or CrkL siRNAs, depending on the experimental conditions. Transduced or transfected osteoblasts were cultured in α-MEM, supplemented with 10% FBS. Osteoblast differentiation was induced by culturing cells in OGM, containing BMP2 (100 ng/mL), ascorbic acid (50 µg/mL), and β-glycerophosphate (100 mM) for 3 or 6 days. Cultured cells (3 days) were lysed using osteoblast lysis buffer (50 mM Tris-HCl (pH 7.4), 1% Triton X-100, 150 mM NaCl, and 1 mM EDTA), and then these lysates were incubated with p-nitrophenyl phosphate substrate (Sigma-Aldrich) for 10 min at room temperature, before their ALP activity was evaluated at 405 nm using a spectrophotometer. Cultured cells (6 days) were fixed with 70% ethanol and stained with 40 mM alizarin red (pH 4.2) for 10 min at room temperature, to visualize calcium deposits. After washing with phosphate-buffered saline to remove the nonspecific staining, alizarin red-stained cells were visualized with CanoScan 4400F (Canon Inc., Tokyo, Japan) and extracted using a 10% cetylpyridinium chloride solution for 30 min at room temperature. Intensities of the extracts were quantified at 562 nm via densitometry.

### 4.4. Luciferase Assay

HEK-293T cells were plated in 24-well plates at a density of 5 × 10^4^ cells/well in Dulbecco’s modified Eagle’s medium (HyClone), supplemented with 10% FBS. The cells were then transfected with reporter luciferase plasmids expressing or not expressing Runx2 or CrkL using FuGENE 6 (Promega, Madison, WI, USA), according to the manufacturer’s instructions. After 48 h, the cells were lysed using 200 µL of passive lysis buffer (Promega), and the cell lysates were evaluated for luciferase activity using a dual-luciferase reporter assay system (Promega) according to the manufacturer’s protocol. Each of these evaluations were completed in triplicate.

### 4.5. Quantitative Real-Time PCR Analysis

Quantitative real-time PCR analysis was performed in triplicate with Rotor-Gene Q (Qiagen, GmbH, Hilden, Germany) and SYBR Green (Qiagen). The thermal cycling conditions were as follows: 15 min at 95 °C, followed by 40 cycles at 94 °C for 15 s, 58 °C for 30 s, and 72 °C for 30 s. The amount of mRNA was normalized to the expression level of an endogenous housekeeping gene encoding glyceraldehyde-3-phosphate dehydrogenase (*Gapdh*). The relative value for the expression of each target gene, compared to that of the calibrator for that target gene, was expressed as 2^−(Ct-Cc)^ (where Ct and Cc are the mean threshold cycle differences of the target gene and the calibrator gene, respectively, after normalization to the expression level of *Gapdh*). The primer sequences were as follows: *Gapdh*, 5′-TGA CCA CAG TCC ATG CCA TCA CTG-3′ and 5′-CAG GAG ACA ACC TGG TCC TCA GTG-3′; *Crkl*, 5′-GTG TCT CGC ACT ACA TCA TCA A-3′ and 5′-GCT GAG ACA GAA CCC ACT GG-3′; *Runx2*, 5′-CCC AGC CAC CTT TAC CTA CA-3′ and 5′-CAG CGT CAA CAC CAT CAT TC-3′; *Alpl*, 5′-CAA GGA TAT CGA CGT GAT CAT G-3′ and 5′-GTC AGT CAG GTT GTT CCG ATT C-3′; *Ibsp*, 5′-GGA AGA GGA GAC TTC AAA CGA AG-3′ and 5′-CAT CCA CTT CTG CTT CTT CGT TC-3′; *Tnfsf11*, 5′-CCT GAG ACT CCA TGA AAA CGC-3′ and 5′-TCG CTG GGC CAC ATC CAA CCA TGA-3′; and *Tnfrsf11b*, 5′-CAG TGA GAG TGTGTGTAT TGC AG-3′ and 5′ -TTA TAC AGG GTG CTT TCG ATG AAG -3′.

### 4.6. Retroviral Infection

The packaging cell line Plat-E was transfected with retroviral vectors using FuGENE 6 (Promega), according to the manufacturer’s protocol. The retroviral supernatant was collected after 48 h of transfection, and the collected supernatant was used as the medium for culturing BMMs or osteoblasts for 6 h, in the presence of 10 μg/mL polybrene (Sigma-Aldrich). This medium was later replaced with growth or differentiation medium as necessary.

### 4.7. siRNA Transfection

BMMs or osteoblasts were transfected with control siRNA or CrkL siRNA purchased from Dharmacon using Lipofectamine RNAiMAX (Invitrogen, Waltham, MA, USA), according to the manufacturer’s protocol, for 4 h at 37 °C, and thereafter, the medium was replaced with a growth medium or differentiation medium.

### 4.8. In Vivo Experiments

The calvarial bone destruction model was established by injecting the control of CrkL siRNA (30 µL of 20 µM), mixed with 10 µL of Lipofectamine RNAiMAX (Invitrogen) into the mice calvaria 1 day prior to the implantation of the collagen sponge. On the next day, a collagen sponge treated with PBS or RANKL (2 mg/kg) was implanted onto the calvariae of the mice. Thereafter, control or CrkL siRNA was injected into the calvariae of mice from post-sponge implant from days 1–5 with 2 day intervals. Mice were euthanized and their calvariae were obtained for µCT analyses using a high-resolution Skyscan 1172 system (Skyscan, Kontich, Belgium) at 50 kV and 201 μA, with a 0.5-mm aluminum filter and a resolution of 17 μm pixel^−1^. All animal experiments were approved by the Chonnam National University Medical School Research Institutional Animal Care and Use Committee and were carried out in accordance with approved guidelines.

### 4.9. Immunoprecipitation and Immunoblotting

HEK-293T cells that were transfected with expression plasmids for 2 days were lysed in NP-40 lysis buffer (150 mM NaCl, 1 mM EDTA, 1% NP-40, 50 mM Tris-HCl (pH 8.0), supplemented with aprotinin and phenylmethylsulfonyl fluoride (PMSF). The lysate was incubated with protein G (Invitrogen) for preclearing, and further incubated overnight with anti-FLAG M2 antibody (Sigma-Aldrich). On the next day, immunoprecipitated lysate was incubated with protein G (Invitrogen), according to the manufacturer’s instructions, and, thereafter, the immunoprecipitated protein or whole cell lysate was subjected to sodium dodecyl sulfate–polyacrylamide gel electrophoresis and polyvinylidene difluoride membrane transfer. The membrane was blocked with 5% skim milk in TBS-T (10 mM Tri-HCl (pH 7.6), 150 mM NaCl, and 0.1% Tween 20), and immunoblotted with primary antibodies against FLAG (Sigma-Aldrich) and Runx2 (Santa Cruz), followed by washing and incubation with appropriate horseradish peroxidase-linked secondary antibodies. Signals were detected with ECL solution (Millipore) and analyzed using an Azure c300 luminescent image analyzer (Azure Biosystems, Dublin, CA, USA).

### 4.10. Statistical Analysis

All values are expressed as the means ± standard deviation (SD). Statistical significance was determined by using two-tailed Student’s *t*-tests for two independent samples, or analysis of variance (ANOVA) with post hoc Tukey’s HSD test for multiple group comparisons. *p* values less than 0.05 were considered as statistically significant.

## 5. Conclusions

CrkL inhibits BMP2-mediated osteoblast differentiation of the osteoblast precursors, while enhancing RANKL-induced osteoclast differentiation of the osteoclast precursors. CrkL also affects both osteoblast-dependent osteoclast differentiation and osteoclast-dependent osteoblast differentiation via its suppression of RANKL expression in osteoblasts. Further evaluations of the in vivo roles of CrkL during bone remodeling will help to understand the potential therapeutic value of CrkL in the future treatment of various bone diseases.

## Data Availability

All data generated or analyzed during this study are in this published article.

## Abbreviations

ALP	alkaline phosphatase
BMMs	bone marrow-derived monocyte/macrophages lineage cells
BMP2	bone morphogenic protein 2
BSP	bone sialoprotein
CrkL	v-crk avian sarcoma virus CT10 oncogene homolog-like
M-CSF	macrophage colony-stimulating factor
OGM	osteogenic media
OPG	osteoprotegerin
PGE_2_	prostaglandin E_2_
RANK	receptor activator of nuclear factor kappa-B
RANKL	receptor activator of nuclear factor kappa-B ligand
Runx2	runt-related transcription factor 2
TRAP	tartrate-resistant acid phosphatase
Vit D_3_	1,25 (OH)_2_ vitamin D_3_

## References

[1] Walsh M.C., Kim N., Kadono Y., Rho J., Lee S.Y., Lorenzo J., Choi Y. (2006). Osteoimmunology: Interplay between the immune system and bone metabolism. Annu. Rev. Immunol..

[2] Cappariello A., Maurizi A., Veeriah V., Teti A. (2014). The Great Beauty of the osteoclast. Arch. Biochem. Biophys..

[3] Kim J.H., Kim N. (2016). Signaling Pathways in Osteoclast Differentiation. Chonnam Med. J..

[4] Kim B.J., Koh J.M. (2019). Coupling factors involved in preserving bone balance. Cell. Mol. Life Sci..

[5] Rodan G.A., Martin T.J. (1981). Role of osteoblasts in hormonal control of bone resorption—A hypothesis. Calcif. Tissue Int..

[6] Tamma R., Zallone A. (2012). Osteoblast and osteoclast crosstalks: From OAF to Ephrin. Inflamm. Allergy Drug Targets.

[7] Mundy G.R., Eleftesriou F. (2006). Boning up on ephrin signaling. Cell.

[8] Sambandam Y., Blanchard J.J., Daughtridge G., Kolb R.J., Shanmugarajan S., Pandruvada S.N., Bateman T.A., Reddy S.V. (2010). Microarray profile of gene expression during osteoclast differentiation in modelled microgravity. J. Cell. Biochem..

[9] Anderson D.M., Maraskovsky E., Billingsley W.L., Dougall W.C., Tometsko M.E., Roux E.R., Teepe M.C., DuBose R.F., Cosman D., Galibert L. (1997). A homologue of the TNF receptor and its ligand enhance T-cell growth and dendritic-cell function. Nature.

[10] Wong B.R., Rho J., Arron J., Robinson E., Orlinick J., Chao M., Kalachikov S., Cayani E., Bartlett F.S., Frankel W.N. (1997). TRANCE is a novel ligand of the tumor necrosis factor receptor family that activates c-Jun N-terminal kinase in T cells. J. Biol. Chem..

[11] Wang L., Liu S., Zhao Y., Liu D., Liu Y., Chen C., Karray S., Shi S., Jin Y. (2015). Osteoblast-induced osteoclast apoptosis by fas ligand/FAS pathway is required for maintenance of bone mass. Cell Death Differ..

[12] Matsuoka K., Park K.A., Ito M., Ikeda K., Takeshita S. (2014). Osteoclast-derived complement component 3a stimulates osteoblast differentiation. J. Bone Miner. Res..

[13] Negishi-Koga T., Shinohara M., Komatsu N., Bito H., Kodama T., Friedel R.H., Takayanagi H. (2011). Suppression of bone formation by osteoclastic expression of semaphorin 4D. Nat. Med..

[14] Zhang Y., Wei L., Miron R.J., Shi B., Bian Z. (2015). Anabolic bone formation via a site-specific bone-targeting delivery system by interfering with semaphorin 4D expression. J. Bone Miner. Res..

[15] Chen X., Wang Z., Duan N., Zhu G., Schwarz E.M., Xie C. (2018). Osteoblast-osteoclast interactions. Connect. Tissue Res..

[16] Yuan F.L., Wu Q.Y., Miao Z.N., Xu M.H., Xu R.S., Jiang D.L., Ye J.X., Chen F.H., Zhao M.D., Wang H.J. (2018). Osteoclast-Derived Extracellular Vesicles: Novel Regulators of Osteoclastogenesis and Osteoclast-Osteoblasts Communication in Bone Remodeling. Front. Physiol..

[17] Sims N.A., Martin T.J. (2015). Coupling Signals between the Osteoclast and Osteoblast: How are Messages Transmitted between These Temporary Visitors to the Bone Surface?. Front. Endocrinol..

[18] Chang B., Quan Q., Li Y., Qiu H., Peng J., Gu Y. (2018). Treatment of Osteoporosis, with a Focus on 2 Monoclonal Antibodies. Med Sci. Monit..

[19] Qaseem A., Forciea M.A., McLean R.M., Denberg T.D. (2017). Treatment of Low Bone Density or Osteoporosis to Prevent Fractures in Men and Women: A Clinical Practice Guideline Update From the American College of Physicians. Ann. Intern. Med..

[20] Cotts K.G., Cifu A.S. (2018). Treatment of Osteoporosis. JAMA.

[21] Rachner T.D., Khosla S., Hofbauer L.C. (2011). Osteoporosis: Now and the future. Lancet.

[22] Cosman F., de Beur S.J., LeBoff M.S., Lewiecki E.M., Tanner B., Randall S., Lindsay R. (2014). Clinician’s Guide to Prevention and Treatment of Osteoporosis. Osteoporos Int..

[23] Shigeno-Nakazawa Y., Kasai T., Ki S., Kostyanovskaya E., Pawlak J., Yamagishi J., Okimoto N., Taiji M., Okada M., Westbrook J. (2016). A pre-metazoan origin of the CRK gene family and co-opted signaling network. Sci. Rep..

[24] Roy N.H., Mammadli M., Burkhardt J.K., Karimi M. (2020). CrkL is required for donor T cell migration to GvHD target organs. Oncotarget.

[25] Song Q., Yi F., Zhang Y., Li D.K.J., Wei Y., Yu H., Zhang Y. (2019). CRKL regulates alternative splicing of cancer-related genes in cervical cancer samples and HeLa cell. BMC Cancer.

[26] Birge R.B., Kalodimos C., Inagaki F., Tanaka S. (2009). Crk and CrkL adaptor proteins: Networks for physiological and pathological signaling. Cell Commun. Signal..

[27] Ren R., Ye Z.S., Baltimore D. (1994). Abl protein-tyrosine kinase selects the Crk adapter as a substrate using SH3-binding sites. Genes Dev..

[28] Sakai R., Iwamatsu A., Hirano N., Ogawa S., Tanaka T., Mano H., Yazaki Y., Hirai H. (1994). A novel signaling molecule, p130, forms stable complexes in vivo with v-Crk and v-Src in a tyrosine phosphorylation-dependent manner. EMBO J..

[29] De Jong R., ten Hoeve J., Heisterkamp N., Groffen J. (1995). Crkl is complexed with tyrosine-phosphorylated Cbl in Ph-positive leukemia. J. Biol. Chem..

[30] Salgia R., Uemura N., Okuda K., Li J.L., Pisick E., Sattler M., de Jong R., Druker B., Heisterkamp N., Chen L.B. (1995). CRKL links p210BCR/ABL with paxillin in chronic myelogenous leukemia cells. J. Biol. Chem..

[31] Salgia R., Pisick E., Sattler M., Li J.L., Uemura N., Wong W.K., Burky S.A., Hirai H., Chen L.B., Griffin J.D. (1996). p130CAS forms a signaling complex with the adapter protein CRKL in hematopoietic cells transformed by the BCR/ABL oncogene. J. Biol. Chem..

[32] Ribon V., Hubbell S., Herrera R., Saltiel A.R. (1996). The product of the cbl oncogene forms stable complexes in vivo with endogenous Crk in a tyrosine phosphorylation-dependent manner. Mol. Cell. Biol..

[33] Beitner-Johnson D., Blakesley V.A., Shen-Orr Z., Jimenez M., Stannard B., Wang L.M., Pierce J., LeRoith D. (1996). The proto-oncogene product c-Crk associates with insulin receptor substrate-1 and 4PS. Modulation by insulin growth factor-I (IGF) and enhanced IGF-I signaling. J. Biol. Chem..

[34] Sattler M., Salgia R., Okuda K., Uemura N., Durstin M.A., Pisick E., Xu G., Li J.L., Prasad K.V., Griffin J.D. (1996). The proto-oncogene product p120CBL and the adaptor proteins CRKL and c-CRK link c-ABL, p190BCR/ABL and p210BCR/ABL to the phosphatidylinositol-3’ kinase pathway. Oncogene.

[35] Akagi T., Shishido T., Murata K., Hanafusa H. (2000). v-Crk activates the phosphoinositide 3-kinase/AKT pathway in transformation. Proc. Natl. Acad. Sci. USA.

[36] Sattler M., Salgia R., Shrikhande G., Verma S., Uemura N., Law S.F., Golemis E.A., Griffin J.D. (1997). Differential signaling after beta1 integrin ligation is mediated through binding of CRKL to p120(CBL) and p110(HEF1). J. Biol. Chem..

[37] Sattler M., Salgia R., Shrikhande G., Verma S., Pisick E., Prasad K.V., Griffin J.D. (1997). Steel factor induces tyrosine phosphorylation of CRKL and binding of CRKL to a complex containing c-kit, phosphatidylinositol 3-kinase, and p120(CBL). J. Biol. Chem..

[38] Gesbert F., Garbay C., Bertoglio J. (1998). Interleukin-2 stimulation induces tyrosine phosphorylation of p120-Cbl and CrkL and formation of multimolecular signaling complexes in T lymphocytes and natural killer cells. J. Biol. Chem..

[39] Koval A.P., Karas M., Zick Y., LeRoith D. (1998). Interplay of the proto-oncogene proteins CrkL and CrkII in insulin-like growth factor-I receptor-mediated signal transduction. J. Biol. Chem..

[40] Fish E.N., Uddin S., Korkmaz M., Majchrzak B., Druker B.J., Platanias L.C. (1999). Activation of a CrkL-stat5 signaling complex by type I interferons. J. Biol. Chem..

[41] Kim J.H., Kim K., Kim I., Seong S., Nam K.I., Kim K.K., Kim N. (2019). Adaptor protein CrkII negatively regulates osteoblast differentiation and function through JNK phosphorylation. Exp. Mol. Med..

[42] Kim J.H., Kim K., Kim I., Seong S., Nam K.I., Lee S.H., Kim K.K., Kim N. (2016). Role of CrkII Signaling in RANKL-Induced Osteoclast Differentiation and Function. J. Immunol..

[43] Guris D.L., Fantes J., Tara D., Druker B.J., Imamoto A. (2001). Mice lacking the homologue of the human 22q11.2 gene CRKL phenocopy neurocristopathies of DiGeorge syndrome. Nat. Genet..

[44] Park T.J., Boyd K., Curran T. (2006). Cardiovascular and craniofacial defects in Crk-null mice. Mol. Cell Biol..

[45] Ikebuchi Y., Aoki S., Honma M., Hayashi M., Sugamori Y., Khan M., Kariya Y., Kato G., Tabata Y., Penninger J.M. (2018). Coupling of bone resorption and formation by RANKL reverse signalling. Nature.

[46] Ozaki Y., Koide M., Furuya Y., Ninomiya T., Yasuda H., Nakamura M., Kobayashi Y., Takahashi N., Yoshinari N., Udagawa N. (2017). Treatment of OPG-deficient mice with WP9QY, a RANKL-binding peptide, recovers alveolar bone loss by suppressing osteoclastogenesis and enhancing osteoblastogenesis. PLoS ONE.

[47] Sawa M., Wakitani S., Kamei N., Kotaka S., Adachi N., Ochi M. (2018). Local administration of WP9QY (W9) peptide promotes bone formation in a rat femur delayed-union model. J. Bone Miner. Metab..

[48] Otsuki Y., Ii M., Moriwaki K., Okada M., Ueda K., Asahi M. (2018). W9 peptide enhanced osteogenic differentiation of human adipose-derived stem cells. Biochem. Biophys. Res. Commun..

[49] Geoffroy V., Kneissel M., Fournier B., Boyde A., Matthias P. (2002). High bone resorption in adult aging transgenic mice overexpressing cbfa1/runx2 in cells of the osteoblastic lineage. Mol. Cell. Biol..

[50] Enomoto H., Shiojiri S., Hoshi K., Furuichi T., Fukuyama R., Yoshida C.A., Kanatani N., Nakamura R., Mizuno A., Zanma A. (2003). Induction of osteoclast differentiation by Runx2 through receptor activator of nuclear factor-kappa B ligand (RANKL) and osteoprotegerin regulation and partial rescue of osteoclastogenesis in Runx2−/− mice by RANKL transgene. J. Biol. Chem..

[51] Yahiro Y., Maeda S., Morikawa M., Koinuma D., Jokoji G., Ijuin T., Komiya S., Kageyama R., Miyazono K., Taniguchi N. (2020). BMP-induced Atoh8 attenuates osteoclastogenesis by suppressing Runx2 transcriptional activity and reducing the Rankl/Opg expression ratio in osteoblasts. Bone Res..

[52] Byon C.H., Sun Y., Chen J., Yuan K., Mao X., Heath J.M., Anderson P.G., Tintut Y., Demer L.L., Wang D. (2011). Runx2-upregulated receptor activator of nuclear factor κB ligand in calcifying smooth muscle cells promotes migration and osteoclastic differentiation of macrophages. Arterioscler. Thromb. Vasc. Biol..

[53] Komori T., Yagi H., Nomura S., Yamaguchi A., Sasaki K., Deguchi K., Shimizu Y., Bronson R.T., Gao Y.H., Inada M. (1997). Targeted disruption of Cbfa1 results in a complete lack of bone formation owing to maturational arrest of osteoblasts. Cell.

[54] Fakhry M., Hamade E., Badran B., Buchet R., Magne D. (2013). Molecular mechanisms of mesenchymal stem cell differentiation towards osteoblasts. World J. Stem Cells.

[55] Mori K., Kitazawa R., Kondo T., Maeda S., Yamaguchi A., Kitazawa S. (2006). Modulation of mouse RANKL gene expression by Runx2 and PKA pathway. J. Cell. Biochem..

[56] O’Brien C.A., Kern B., Gubrij I., Karsenty G., Manolagas S.C. (2002). Cbfa1 does not regulate RANKL gene activity in stromal/osteoblastic cells. Bone.

